# 1-Benzoyl-3,3-bis­(propan-2-yl)thio­urea

**DOI:** 10.1107/S1600536810028862

**Published:** 2010-07-24

**Authors:** N. Gunasekaran, R. Karvembu, Seik Weng Ng, Edward R. T. Tiekink

**Affiliations:** a Department of Chemistry, National Institute of Technology, Tiruchirappalli 620 015, India; b Department of Chemistry, University of Malaya, 50603 Kuala Lumpur, Malaysia

## Abstract

Two independent thio­urea derivatives comprise the asymmetric unit of the title compound, C_14_H_20_N_2_OS. The major difference between the mol­ecules relates to a twist in the relative orientation of the benzene rings [torsion angles = 4.5 (2) and −19.9 (2)° for the two independent mol­ecules]. The thio­carbonyl and carbonyl groups lie to opposite sides of the mol­ecule as there are twists about the central N—S bond [torsion angles = 83.90 (15) and 81.77 (15)°]. Supra­molecular chains extending parallel to [101] with a stepped topology and mediated by N—H⋯O hydrogen bonding feature in the crystal structure. C—H⋯O and C—H⋯π inter­actions are also present.

## Related literature

For the biological activity of thio­urea derivatives, see: Venkatachalam *et al.* (2004 ▶); Yuan *et al.* (2001 ▶); Zhou *et al.* (2004 ▶). For the use of ruthenium(III) complexes of thio­ureas as catalysts, see: Gunasekaran & Karvembru (2010 ▶). For additional structural analysis, see: Spek (2009 ▶).
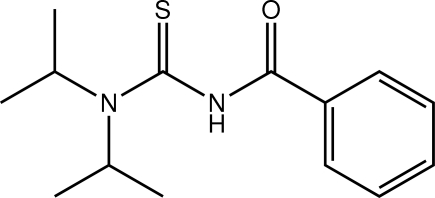


## Experimental

### 

#### Crystal data

C_14_H_20_N_2_OS
*M* *_r_* = 264.38
Monoclinic, 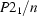

*a* = 14.8072 (10) Å
*b* = 13.5832 (10) Å
*c* = 14.9168 (11) Å
β = 97.635 (1)°
*V* = 2973.6 (4) Å^3^
*Z* = 8
Mo *K*α radiation
μ = 0.21 mm^−1^
*T* = 100 K
0.40 × 0.25 × 0.05 mm

#### Data collection

Bruker SMART APEX diffractometer
Absorption correction: multi-scan (*SADABS*; Sheldrick, 1996 ▶) *T*_min_ = 0.921, *T*_max_ = 0.990
27965 measured reflections
6833 independent reflections
5254 reflections with *I* > 2σ(*I*)
*R*_int_ = 0.051

#### Refinement

*R*[*F* ^2^ > 2σ(*F* ^2^)] = 0.037
*wR*(*F* ^2^) = 0.097
*S* = 1.02
6833 reflections
341 parameters
2 restraints
H atoms treated by a mixture of independent and constrained refinement
Δρ_max_ = 0.32 e Å^−3^
Δρ_min_ = −0.22 e Å^−3^

### 

Data collection: *APEX2* (Bruker, 2008 ▶); cell refinement: *SAINT* (Bruker, 2008 ▶); data reduction: *SAINT*; program(s) used to solve structure: *SHELXS97* (Sheldrick, 2008 ▶); program(s) used to refine structure: *SHELXL97* (Sheldrick, 2008 ▶); molecular graphics: *ORTEP-3* (Farrugia, 1997 ▶), *DIAMOND* (Brandenburg, 2006 ▶) and *Qmol* (Gans & Shalloway, 2001 ▶); software used to prepare material for publication: *publCIF* (Westrip, 2010 ▶).

## Supplementary Material

Crystal structure: contains datablocks global, I. DOI: 10.1107/S1600536810028862/ez2225sup1.cif

Structure factors: contains datablocks I. DOI: 10.1107/S1600536810028862/ez2225Isup2.hkl

Additional supplementary materials:  crystallographic information; 3D view; checkCIF report

## Figures and Tables

**Table 1:** Hydrogen-bond geometry (Å, °) *Cg* is the centroid of the C1–C6 ring.

*D*—H⋯*A*	*D*—H	H⋯*A*	*D*⋯*A*	*D*—H⋯*A*
N1—H1⋯O2^i^	0.85 (1)	2.03 (1)	2.8568 (16)	167 (2)
N3—H3⋯O1	0.85 (1)	1.94 (1)	2.7728 (16)	163 (2)
C6—H6⋯O2^i^	0.95	2.40	3.3306 (18)	166
C10—H10c⋯*Cg*^ii^	0.98	2.63	3.5714 (18)	160
C25—H25b⋯*Cg*^iii^	0.98	2.70	3.5717 (18)	149

Symmetry codes: (i) 
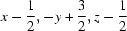
; (ii) 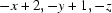; (iii) 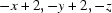.

**Fig. 1.**
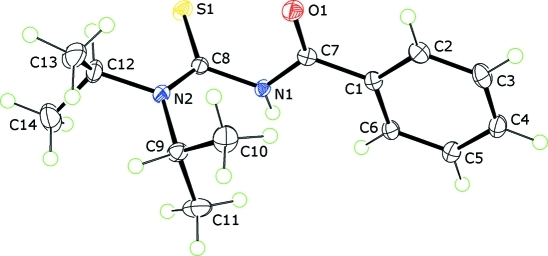
The molecular structure of the first independent molecule in (I) showing the atom-labelling scheme and displacement ellipsoids at the 50% probability level.

**Fig. 2.**
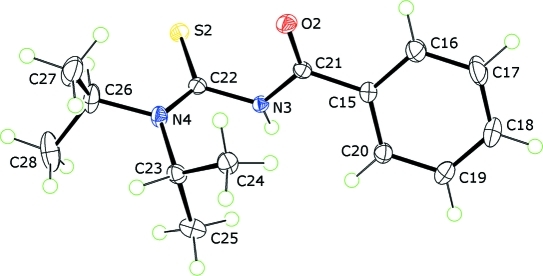
The molecular structure of the second independent molecule in (I) showing the atom-labelling scheme and displacement ellipsoids at the 50% probability level.

**Fig. 3.**
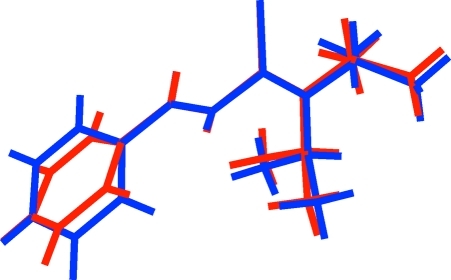
Overlay diagram of the first independent molecule (shown in red) and the second independent molecule (shown in blue).

**Fig. 4.**
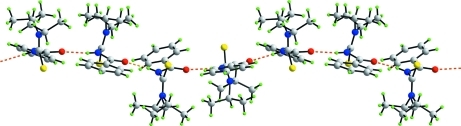
Linear supramolecular chain along the 1 0 1 direction in (I) mediated by N–H···O hydrogen bonding, shown as orange dashed lines.

**Fig. 5.**
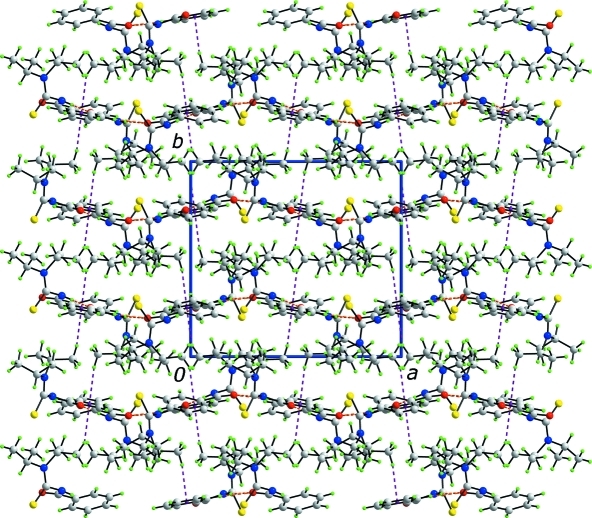
Unit-cell contents shown in projection down the c axis in (I). The N–H···O hydrogen bonding and C–H···π contacts are shown as orange and purple dashed lines, respectively.

## supplementary crystallographic information

### Comment

Thiourea and its derivatives are useful as anti-tumour, anti-fungal, anti-bacterial, insecticidal, herbicidal, pesticidal agents, and plant-growth regulators (Venkatachalam *et al.*, 2004; Yuan *et al.*, 2001; Zhou *et al.*, 2004). *N*-[Di(alkyl/aryl)carbamothioyl]benzamide derivatives provide immense opportunities for altering electronic and steric effects in its metal complexes. This might help in designing effective catalysts. Ruthenium(III) complexes containing these ligands have recently been used as catalysts for oxidation of alcohols to carbonyl compounds (Gunasekaran *et al.*, 2010). The structure of the title thiourea derivative, (I), was investigated to provide reference data for subsequent studies.

Two independent molecules comprise the asymmetric unit of (I). The first independent molecule, Fig. 1, is virtually super-imposable upon the second, Fig. 2, with the major difference between them being a twist in the benzene rings, Fig. 3. This is quantified by the C2–C1–C7–O1 and C16–C15–C21–O2 torsion angles of 4.5 (2) and -19.9 (2) °, respectively. This is also reflected in the r.m.s. deviation for bond distances and angles of 0.0035 Å and 0.903 °, respectively (Spek, 2009). The molecules are twisted about the central thiourea bond as seen in the C7—N1—C8—S1 and C21—N3—C22—S2 torsion angles of 83.90 (15) and 81.77 (15) °, respectively, indicating that the thiocarbonyl and carbonyl groups lie to opposite sides of the molecule.

The most notable feature in the crystal packing is the formation of supramolecular chains mediated by N–H···O hydrogen bonding, Table 1; chains are reinforced by C–H···O contacts involving the O2 atom, Table 1. The chains comprise alternating pairs of molecules of opposite orientation so that the topology is stepped. Chains aggregate into layers in the *ac* plane with the primary interactions between them along the *b* axis being of the type C–H···π, Fig. 5 and Table 1.

### Experimental

A solution of benzoyl chloride (0.70285 g, 5 mmol) in acetone (50 ml) was added drop wise to a suspension of potassium thiocyanate (0.4859 g, 5 mmol) in anhydrous acetone (50 ml). The reaction mixture was heated under reflux for 45 minutes and then cooled to room temperature. A solution of diisopropyl amine (0.5059 g, 5 mmol) in acetone (30 ml) was added and the resulting mixture was stirred for 2 h. Hydrochloric acid (0.1 N, 300 ml) was added and the resulting white solid was filtered, washed with water and dried *in vacuo*. Single crystals for X-ray diffraction were grown at room temperature from ethyl acetate solution by the diffusion of diethyl ether vapour. Yield 78%; *M*. Pt. 383 K; FT—IR (KBr) ν(N—H) 3249, ν(C═O) 1651, ν(C═ S) 1279 cm^-1^.

### Refinement

Carbon-bound H-atoms were placed in calculated positions (C—H 0.95 to 1.00 Å) and were included in the refinement in the riding model approximation, with *U*_iso_(H) set to 1.2 to 1.5*U*_equiv_(C). The N-bound H-atoms were located in a difference Fourier map, and were refined with a distance restraint of N–H 0.86±0.01 Å; their *U_iso_* values were freely refined

### Crystal data

**Table:**

C_14_H_20_N_2_OS	*F*(000) = 1136
*M**_r_* = 264.38	*D*_x_ = 1.181 Mg m^−^^3^
Monoclinic, *P*2_1_/*n*	Mo *K*α radiation, λ = 0.71073 Å
Hall symbol: -P 2yn	Cell parameters from 5840 reflections
*a* = 14.8072 (10) Å	θ = 2.4–28.1°
*b* = 13.5832 (10) Å	µ = 0.21 mm^−^^1^
*c* = 14.9168 (11) Å	*T* = 100 K
β = 97.635 (1)°	Prism, colourless
*V* = 2973.6 (4) Å^3^	0.40 × 0.25 × 0.05 mm
*Z* = 8	

### Data collection

**Table:**

Bruker SMART APEX diffractometer	6833 independent reflections
Radiation source: fine-focus sealed tube	5254 reflections with *I* > 2σ(*I*)
graphite	*R*_int_ = 0.051
ω scans	θ_max_ = 27.5°, θ_min_ = 1.8°
Absorption correction: multi-scan (*SADABS*; Sheldrick, 1996)	*h* = −19→19
*T*_min_ = 0.921, *T*_max_ = 0.990	*k* = −17→17
27965 measured reflections	*l* = −19→19

### Refinement

**Table:**

Refinement on *F*^2^	Primary atom site location: structure-invariant direct methods
Least-squares matrix: full	Secondary atom site location: difference Fourier map
*R*[*F*^2^ > 2σ(*F*^2^)] = 0.037	Hydrogen site location: inferred from neighbouring sites
*wR*(*F*^2^) = 0.097	H atoms treated by a mixture of independent and constrained refinement
*S* = 1.02	*w* = 1/[σ^2^(*F*_o_^2^) + (0.0476*P*)^2^ + 0.4491*P*] where *P* = (*F*_o_^2^ + 2*F*_c_^2^)/3
6833 reflections	(Δ/σ)_max_ = 0.001
341 parameters	Δρ_max_ = 0.32 e Å^−^^3^
2 restraints	Δρ_min_ = −0.22 e Å^−^^3^

### Special details

**Table:**

Geometry. All e.s.d.'s (except the e.s.d. in the dihedral angle between two l.s. planes) are estimated using the full covariance matrix. The cell e.s.d.'s are taken into account individually in the estimation of e.s.d.'s in distances, angles and torsion angles; correlations between e.s.d.'s in cell parameters are only used when they are defined by crystal symmetry. An approximate (isotropic) treatment of cell e.s.d.'s is used for estimating e.s.d.'s involving l.s. planes.
Refinement. Refinement of *F*^2^ against ALL reflections. The weighted *R*-factor *wR* and goodness of fit *S* are based on *F*^2^, conventional *R*-factors *R* are based on *F*, with *F* set to zero for negative *F*^2^. The threshold expression of *F*^2^ > σ(*F*^2^) is used only for calculating *R*-factors(gt) *etc*. and is not relevant to the choice of reflections for refinement. *R*-factors based on *F*^2^ are statistically about twice as large as those based on *F*, and *R*- factors based on ALL data will be even larger.

### Fractional atomic coordinates and isotropic or equivalent isotropic displacement parameters (Å^2^)

**Table:**

	*x*	*y*	*z*	*U*_iso_*/*U*_eq_	
S1	0.75721 (3)	0.79767 (3)	0.15341 (3)	0.01907 (10)	
S2	1.24751 (3)	0.74064 (3)	0.16642 (3)	0.01868 (10)	
O1	0.97620 (7)	0.75101 (8)	0.11338 (7)	0.0217 (2)	
O2	1.20465 (7)	0.80331 (8)	0.38108 (7)	0.0198 (2)	
N1	0.84390 (8)	0.70403 (9)	0.03311 (8)	0.0135 (2)	
H1	0.8091 (11)	0.7049 (14)	−0.0167 (8)	0.033 (5)*	
N2	0.80562 (8)	0.60687 (9)	0.15068 (8)	0.0160 (3)	
N3	1.11221 (8)	0.81185 (9)	0.24783 (8)	0.0136 (3)	
H3	1.0643 (9)	0.7908 (14)	0.2154 (12)	0.037 (6)*	
N4	1.19743 (9)	0.92929 (9)	0.18289 (8)	0.0172 (3)	
C1	0.97327 (10)	0.74376 (10)	−0.04643 (10)	0.0150 (3)	
C2	1.06667 (10)	0.76249 (11)	−0.03733 (11)	0.0191 (3)	
H2	1.0994	0.7740	0.0209	0.023*	
C3	1.11180 (11)	0.76431 (11)	−0.11299 (11)	0.0213 (3)	
H3A	1.1754	0.7772	−0.1063	0.026*	
C4	1.06483 (11)	0.74753 (11)	−0.19821 (11)	0.0202 (3)	
H4	1.0961	0.7478	−0.2498	0.024*	
C5	0.97160 (10)	0.73027 (11)	−0.20766 (10)	0.0183 (3)	
H5	0.9390	0.7197	−0.2662	0.022*	
C6	0.92549 (10)	0.72824 (10)	−0.13246 (10)	0.0164 (3)	
H6	0.8617	0.7163	−0.1395	0.020*	
C7	0.93123 (10)	0.73556 (10)	0.03927 (10)	0.0153 (3)	
C8	0.80264 (10)	0.69632 (10)	0.11469 (9)	0.0142 (3)	
C9	0.83729 (11)	0.51575 (10)	0.10756 (10)	0.0188 (3)	
H9	0.8239	0.4604	0.1480	0.023*	
C10	0.93975 (11)	0.51274 (12)	0.10634 (12)	0.0270 (4)	
H10A	0.9708	0.5373	0.1641	0.041*	
H10B	0.9557	0.5542	0.0570	0.041*	
H10C	0.9588	0.4448	0.0972	0.041*	
C11	0.78275 (13)	0.49311 (12)	0.01598 (11)	0.0295 (4)	
H11A	0.7175	0.4995	0.0203	0.044*	
H11B	0.7959	0.4258	−0.0020	0.044*	
H11C	0.7997	0.5395	−0.0292	0.044*	
C12	0.77042 (12)	0.59103 (12)	0.23844 (11)	0.0252 (4)	
H12	0.7585	0.6574	0.2633	0.030*	
C13	0.84165 (14)	0.54090 (13)	0.30663 (11)	0.0340 (4)	
H13A	0.8201	0.5393	0.3660	0.051*	
H13B	0.8990	0.5777	0.3111	0.051*	
H13C	0.8516	0.4735	0.2867	0.051*	
C14	0.68014 (13)	0.53676 (13)	0.22384 (14)	0.0364 (5)	
H14A	0.6374	0.5727	0.1798	0.055*	
H14B	0.6552	0.5321	0.2813	0.055*	
H14C	0.6896	0.4704	0.2010	0.055*	
C15	1.04692 (10)	0.77094 (10)	0.38361 (10)	0.0162 (3)	
C16	1.06181 (12)	0.72906 (13)	0.46947 (11)	0.0253 (4)	
H16	1.1218	0.7118	0.4953	0.030*	
C17	0.98915 (13)	0.71256 (14)	0.51739 (12)	0.0326 (4)	
H17	0.9994	0.6828	0.5755	0.039*	
C18	0.90163 (12)	0.73933 (13)	0.48099 (12)	0.0290 (4)	
H18	0.8522	0.7297	0.5147	0.035*	
C19	0.88646 (11)	0.78013 (12)	0.39528 (11)	0.0237 (4)	
H19	0.8264	0.7980	0.3701	0.028*	
C20	0.95841 (10)	0.79518 (11)	0.34577 (11)	0.0190 (3)	
H20	0.9474	0.8218	0.2864	0.023*	
C21	1.12797 (10)	0.79444 (10)	0.33795 (10)	0.0149 (3)	
C22	1.18641 (10)	0.83429 (10)	0.19877 (9)	0.0143 (3)	
C23	1.13244 (10)	1.00932 (11)	0.20189 (10)	0.0181 (3)	
H23	1.1600	1.0720	0.1831	0.022*	
C24	1.12179 (11)	1.02287 (11)	0.30114 (10)	0.0200 (3)	
H24A	1.0985	1.0891	0.3105	0.030*	
H24B	1.1811	1.0144	0.3382	0.030*	
H24C	1.0789	0.9738	0.3187	0.030*	
C25	1.04171 (11)	0.99951 (12)	0.14111 (11)	0.0256 (4)	
H25A	1.0527	0.9926	0.0781	0.038*	
H25B	1.0047	1.0584	0.1472	0.038*	
H25C	1.0093	0.9413	0.1590	0.038*	
C26	1.27615 (12)	0.96155 (12)	0.13727 (12)	0.0271 (4)	
H26	1.3095	0.9008	0.1228	0.033*	
C27	1.34174 (12)	1.02249 (13)	0.20183 (14)	0.0340 (4)	
H27A	1.3604	0.9847	0.2571	0.051*	
H27B	1.3116	1.0833	0.2170	0.051*	
H27C	1.3956	1.0388	0.1730	0.051*	
C28	1.24534 (15)	1.01339 (15)	0.04833 (13)	0.0446 (5)	
H28A	1.2010	0.9721	0.0109	0.067*	
H28B	1.2981	1.0251	0.0164	0.067*	
H28C	1.2170	1.0764	0.0602	0.067*	

### Atomic displacement parameters (Å^2^)

**Table:**

	*U*^11^	*U*^22^	*U*^33^	*U*^12^	*U*^13^	*U*^23^
S1	0.0237 (2)	0.01671 (18)	0.0177 (2)	0.00430 (15)	0.00614 (16)	−0.00098 (14)
S2	0.0191 (2)	0.01741 (18)	0.0205 (2)	0.00404 (14)	0.00623 (15)	0.00049 (14)
O1	0.0178 (6)	0.0305 (6)	0.0161 (5)	−0.0038 (5)	−0.0002 (4)	−0.0028 (5)
O2	0.0143 (6)	0.0287 (6)	0.0156 (5)	0.0002 (4)	−0.0011 (4)	0.0005 (4)
N1	0.0135 (6)	0.0170 (6)	0.0098 (6)	−0.0006 (5)	0.0010 (5)	0.0005 (5)
N2	0.0200 (7)	0.0146 (6)	0.0144 (6)	−0.0006 (5)	0.0058 (5)	−0.0014 (5)
N3	0.0116 (6)	0.0160 (6)	0.0129 (6)	−0.0011 (5)	0.0007 (5)	0.0003 (5)
N4	0.0200 (7)	0.0154 (6)	0.0175 (6)	−0.0005 (5)	0.0081 (5)	−0.0003 (5)
C1	0.0148 (7)	0.0134 (7)	0.0169 (7)	0.0013 (5)	0.0026 (6)	0.0016 (6)
C2	0.0161 (8)	0.0192 (7)	0.0214 (8)	0.0004 (6)	0.0005 (6)	0.0012 (6)
C3	0.0136 (8)	0.0216 (8)	0.0294 (9)	0.0005 (6)	0.0052 (7)	0.0034 (6)
C4	0.0214 (8)	0.0192 (8)	0.0217 (8)	0.0022 (6)	0.0096 (7)	0.0017 (6)
C5	0.0192 (8)	0.0185 (7)	0.0177 (8)	0.0023 (6)	0.0046 (6)	0.0014 (6)
C6	0.0142 (7)	0.0171 (7)	0.0181 (7)	0.0000 (6)	0.0032 (6)	0.0009 (6)
C7	0.0145 (7)	0.0143 (7)	0.0167 (7)	0.0009 (6)	0.0007 (6)	−0.0002 (6)
C8	0.0116 (7)	0.0181 (7)	0.0126 (7)	−0.0017 (6)	0.0005 (6)	−0.0009 (6)
C9	0.0260 (9)	0.0134 (7)	0.0176 (8)	0.0023 (6)	0.0050 (6)	−0.0008 (6)
C10	0.0286 (10)	0.0216 (8)	0.0323 (10)	0.0071 (7)	0.0090 (8)	0.0028 (7)
C11	0.0416 (11)	0.0195 (8)	0.0256 (9)	0.0008 (7)	−0.0015 (8)	−0.0059 (7)
C12	0.0394 (10)	0.0190 (8)	0.0206 (8)	−0.0025 (7)	0.0172 (7)	0.0002 (6)
C13	0.0594 (13)	0.0255 (9)	0.0180 (8)	−0.0026 (8)	0.0090 (8)	0.0029 (7)
C14	0.0405 (12)	0.0258 (9)	0.0486 (12)	−0.0037 (8)	0.0272 (10)	−0.0002 (8)
C15	0.0172 (8)	0.0164 (7)	0.0155 (7)	−0.0023 (6)	0.0038 (6)	−0.0011 (6)
C16	0.0226 (9)	0.0355 (9)	0.0178 (8)	−0.0006 (7)	0.0027 (7)	0.0034 (7)
C17	0.0370 (11)	0.0463 (11)	0.0158 (8)	−0.0056 (9)	0.0080 (8)	0.0066 (7)
C18	0.0263 (10)	0.0370 (10)	0.0267 (9)	−0.0069 (7)	0.0142 (8)	−0.0026 (7)
C19	0.0176 (8)	0.0266 (8)	0.0282 (9)	−0.0015 (6)	0.0072 (7)	−0.0013 (7)
C20	0.0181 (8)	0.0199 (7)	0.0193 (8)	−0.0015 (6)	0.0039 (6)	0.0010 (6)
C21	0.0163 (8)	0.0135 (7)	0.0149 (7)	0.0011 (6)	0.0015 (6)	0.0000 (5)
C22	0.0133 (7)	0.0182 (7)	0.0108 (7)	−0.0004 (6)	−0.0012 (6)	−0.0007 (5)
C23	0.0218 (8)	0.0140 (7)	0.0189 (8)	0.0022 (6)	0.0045 (6)	0.0010 (6)
C24	0.0249 (9)	0.0167 (7)	0.0193 (8)	0.0016 (6)	0.0067 (7)	−0.0012 (6)
C25	0.0290 (9)	0.0215 (8)	0.0247 (9)	0.0045 (7)	−0.0029 (7)	0.0012 (7)
C26	0.0307 (10)	0.0210 (8)	0.0345 (10)	−0.0038 (7)	0.0225 (8)	−0.0020 (7)
C27	0.0254 (10)	0.0310 (9)	0.0485 (12)	−0.0062 (8)	0.0153 (9)	0.0021 (8)
C28	0.0648 (15)	0.0468 (12)	0.0270 (10)	−0.0187 (10)	0.0240 (10)	0.0011 (9)

### Geometric parameters (Å, °)

**Table:**

S1—C8	1.6687 (15)	C12—C13	1.525 (2)
S2—C22	1.6689 (15)	C12—H12	1.0000
O1—C7	1.2304 (18)	C13—H13A	0.9800
O2—C21	1.2343 (18)	C13—H13B	0.9800
N1—C7	1.3537 (19)	C13—H13C	0.9800
N1—C8	1.4362 (18)	C14—H14A	0.9800
N1—H1	0.847 (9)	C14—H14B	0.9800
N2—C8	1.3266 (18)	C14—H14C	0.9800
N2—C12	1.4877 (18)	C15—C16	1.392 (2)
N2—C9	1.4986 (18)	C15—C20	1.396 (2)
N3—C21	1.3542 (18)	C15—C21	1.491 (2)
N3—C22	1.4316 (18)	C16—C17	1.387 (2)
N3—H3	0.854 (9)	C16—H16	0.9500
N4—C22	1.3260 (18)	C17—C18	1.385 (3)
N4—C26	1.4915 (19)	C17—H17	0.9500
N4—C23	1.5035 (18)	C18—C19	1.384 (2)
C1—C6	1.397 (2)	C18—H18	0.9500
C1—C2	1.395 (2)	C19—C20	1.390 (2)
C1—C7	1.498 (2)	C19—H19	0.9500
C2—C3	1.386 (2)	C20—H20	0.9500
C2—H2	0.9500	C23—C24	1.521 (2)
C3—C4	1.384 (2)	C23—C25	1.523 (2)
C3—H3A	0.9500	C23—H23	1.0000
C4—C5	1.389 (2)	C24—H24A	0.9800
C4—H4	0.9500	C24—H24B	0.9800
C5—C6	1.389 (2)	C24—H24C	0.9800
C5—H5	0.9500	C25—H25A	0.9800
C6—H6	0.9500	C25—H25B	0.9800
C9—C10	1.520 (2)	C25—H25C	0.9800
C9—C11	1.523 (2)	C26—C27	1.519 (3)
C9—H9	1.0000	C26—C28	1.518 (3)
C10—H10A	0.9800	C26—H26	1.0000
C10—H10B	0.9800	C27—H27A	0.9800
C10—H10C	0.9800	C27—H27B	0.9800
C11—H11A	0.9800	C27—H27C	0.9800
C11—H11B	0.9800	C28—H28A	0.9800
C11—H11C	0.9800	C28—H28B	0.9800
C12—C14	1.517 (2)	C28—H28C	0.9800
			
C7—N1—C8	118.40 (12)	C12—C14—H14A	109.5
C7—N1—H1	121.3 (13)	C12—C14—H14B	109.5
C8—N1—H1	117.8 (13)	H14A—C14—H14B	109.5
C8—N2—C12	119.46 (12)	C12—C14—H14C	109.5
C8—N2—C9	125.33 (12)	H14A—C14—H14C	109.5
C12—N2—C9	115.11 (11)	H14B—C14—H14C	109.5
C21—N3—C22	120.13 (12)	C16—C15—C20	119.60 (14)
C21—N3—H3	121.7 (14)	C16—C15—C21	117.98 (14)
C22—N3—H3	114.7 (14)	C20—C15—C21	122.29 (13)
C22—N4—C26	119.31 (12)	C17—C16—C15	120.10 (16)
C22—N4—C23	124.95 (12)	C17—C16—H16	120.0
C26—N4—C23	115.61 (11)	C15—C16—H16	120.0
C6—C1—C2	119.44 (14)	C18—C17—C16	120.32 (16)
C6—C1—C7	123.75 (13)	C18—C17—H17	119.8
C2—C1—C7	116.70 (13)	C16—C17—H17	119.8
C3—C2—C1	120.20 (15)	C19—C18—C17	119.72 (15)
C3—C2—H2	119.9	C19—C18—H18	120.1
C1—C2—H2	119.9	C17—C18—H18	120.1
C4—C3—C2	120.46 (14)	C18—C19—C20	120.53 (16)
C4—C3—H3A	119.8	C18—C19—H19	119.7
C2—C3—H3A	119.8	C20—C19—H19	119.7
C3—C4—C5	119.49 (14)	C19—C20—C15	119.69 (15)
C3—C4—H4	120.3	C19—C20—H20	120.2
C5—C4—H4	120.3	C15—C20—H20	120.2
C6—C5—C4	120.67 (15)	O2—C21—N3	121.71 (13)
C6—C5—H5	119.7	O2—C21—C15	121.55 (13)
C4—C5—H5	119.7	N3—C21—C15	116.60 (13)
C5—C6—C1	119.72 (14)	N4—C22—N3	114.80 (12)
C5—C6—H6	120.1	N4—C22—S2	127.29 (11)
C1—C6—H6	120.1	N3—C22—S2	117.90 (10)
O1—C7—N1	120.86 (14)	N4—C23—C24	115.01 (12)
O1—C7—C1	121.15 (13)	N4—C23—C25	111.13 (12)
N1—C7—C1	117.83 (13)	C24—C23—C25	113.12 (13)
N2—C8—N1	114.50 (12)	N4—C23—H23	105.5
N2—C8—S1	127.50 (11)	C24—C23—H23	105.5
N1—C8—S1	118.00 (10)	C25—C23—H23	105.5
N2—C9—C10	113.40 (12)	C23—C24—H24A	109.5
N2—C9—C11	113.13 (13)	C23—C24—H24B	109.5
C10—C9—C11	113.26 (13)	H24A—C24—H24B	109.5
N2—C9—H9	105.3	C23—C24—H24C	109.5
C10—C9—H9	105.3	H24A—C24—H24C	109.5
C11—C9—H9	105.3	H24B—C24—H24C	109.5
C9—C10—H10A	109.5	C23—C25—H25A	109.5
C9—C10—H10B	109.5	C23—C25—H25B	109.5
H10A—C10—H10B	109.5	H25A—C25—H25B	109.5
C9—C10—H10C	109.5	C23—C25—H25C	109.5
H10A—C10—H10C	109.5	H25A—C25—H25C	109.5
H10B—C10—H10C	109.5	H25B—C25—H25C	109.5
C9—C11—H11A	109.5	N4—C26—C27	110.09 (13)
C9—C11—H11B	109.5	N4—C26—C28	111.89 (15)
H11A—C11—H11B	109.5	C27—C26—C28	113.20 (15)
C9—C11—H11C	109.5	N4—C26—H26	107.1
H11A—C11—H11C	109.5	C27—C26—H26	107.1
H11B—C11—H11C	109.5	C28—C26—H26	107.1
N2—C12—C14	110.44 (14)	C26—C27—H27A	109.5
N2—C12—C13	111.06 (13)	C26—C27—H27B	109.5
C14—C12—C13	113.39 (14)	H27A—C27—H27B	109.5
N2—C12—H12	107.2	C26—C27—H27C	109.5
C14—C12—H12	107.2	H27A—C27—H27C	109.5
C13—C12—H12	107.2	H27B—C27—H27C	109.5
C12—C13—H13A	109.5	C26—C28—H28A	109.5
C12—C13—H13B	109.5	C26—C28—H28B	109.5
H13A—C13—H13B	109.5	H28A—C28—H28B	109.5
C12—C13—H13C	109.5	C26—C28—H28C	109.5
H13A—C13—H13C	109.5	H28A—C28—H28C	109.5
H13B—C13—H13C	109.5	H28B—C28—H28C	109.5
			
C6—C1—C2—C3	−0.9 (2)	C20—C15—C16—C17	−0.7 (2)
C7—C1—C2—C3	175.51 (13)	C21—C15—C16—C17	175.26 (15)
C1—C2—C3—C4	−0.1 (2)	C15—C16—C17—C18	−1.3 (3)
C2—C3—C4—C5	1.0 (2)	C16—C17—C18—C19	1.9 (3)
C3—C4—C5—C6	−1.0 (2)	C17—C18—C19—C20	−0.5 (3)
C4—C5—C6—C1	0.0 (2)	C18—C19—C20—C15	−1.4 (2)
C2—C1—C6—C5	0.9 (2)	C16—C15—C20—C19	2.0 (2)
C7—C1—C6—C5	−175.24 (13)	C21—C15—C20—C19	−173.76 (14)
C8—N1—C7—O1	4.8 (2)	C22—N3—C21—O2	4.6 (2)
C8—N1—C7—C1	−179.69 (12)	C22—N3—C21—C15	−179.58 (12)
C6—C1—C7—O1	−179.23 (14)	C16—C15—C21—O2	−19.9 (2)
C2—C1—C7—O1	4.5 (2)	C20—C15—C21—O2	155.89 (14)
C6—C1—C7—N1	5.2 (2)	C16—C15—C21—N3	164.24 (14)
C2—C1—C7—N1	−171.00 (13)	C20—C15—C21—N3	−19.9 (2)
C12—N2—C8—N1	176.10 (13)	C26—N4—C22—N3	176.26 (13)
C9—N2—C8—N1	−7.7 (2)	C23—N4—C22—N3	−8.0 (2)
C12—N2—C8—S1	−3.9 (2)	C26—N4—C22—S2	−5.0 (2)
C9—N2—C8—S1	172.33 (11)	C23—N4—C22—S2	170.75 (11)
C7—N1—C8—N2	−96.09 (16)	C21—N3—C22—N4	−99.35 (15)
C7—N1—C8—S1	83.90 (15)	C21—N3—C22—S2	81.77 (15)
C8—N2—C9—C10	72.10 (19)	C22—N4—C23—C24	63.67 (19)
C12—N2—C9—C10	−111.54 (15)	C26—N4—C23—C24	−120.46 (15)
C8—N2—C9—C11	−58.6 (2)	C22—N4—C23—C25	−66.49 (18)
C12—N2—C9—C11	117.72 (15)	C26—N4—C23—C25	109.38 (15)
C8—N2—C12—C14	106.66 (16)	C22—N4—C26—C27	−113.95 (16)
C9—N2—C12—C14	−69.93 (17)	C23—N4—C26—C27	69.92 (18)
C8—N2—C12—C13	−126.65 (15)	C22—N4—C26—C28	119.25 (16)
C9—N2—C12—C13	56.76 (17)	C23—N4—C26—C28	−56.87 (18)

### Hydrogen-bond geometry (Å, °)

**Table:**

Cg is the centroid of the C1–C6 ring.

**Table:**

*D*—H···*A*	*D*—H	H···*A*	*D*···*A*	*D*—H···*A*
N1—H1···O2^i^	0.85 (1)	2.03 (1)	2.8568 (16)	167 (2)
N3—H3···O1	0.85 (1)	1.94 (1)	2.7728 (16)	163 (2)
C6—H6···O2^i^	0.95	2.40	3.3306 (18)	166
C10—H10c···Cg^ii^	0.98	2.63	3.5714 (18)	160
C25—H25b···Cg^iii^	0.98	2.70	3.5717 (18)	149

Symmetry codes: (i) *x*−1/2, −*y*+3/2, *z*−1/2; (ii) −*x*+2, −*y*+1, −*z*; (iii) −*x*+2, −*y*+2, −*z*.
